# OA08 A case of Recalcitrant Psoriatic Disease- should new treatment strategies be considered?

**DOI:** 10.1093/rap/rkac066.008

**Published:** 2022-09-28

**Authors:** Sonia Sundanum, Áine Gorman, Douglas Veale

**Affiliations:** Centre for Arthritis and Rheumatic Diseases, Dublin Academic Medical Centre, University College Dublin, Dublin, Ireland; Rheumatology Department, St Vincent's University Hospital, Dublin, Ireland; Centre for Arthritis and Rheumatic Diseases, Dublin Academic Medical Centre, University College Dublin, Dublin, Ireland; Rheumatology Department, St Vincent's University Hospital, Dublin, Ireland; Centre for Arthritis and Rheumatic Diseases, Dublin Academic Medical Centre, University College Dublin, Dublin, Ireland; Rheumatology Department, St Vincent's University Hospital, Dublin, Ireland

## Abstract

**Introduction/Background:**

Despite the advent of numerous biologic therapies and small molecular drugs targeting specific immune pathways, at least 40% of PsA patients exhibit poor response.

Given the complexity and heterogeneity of signalling pathways resulting in synovio-entheseal disease in PsA; new treatment strategies must be evaluated to induce deep and sustainable clinical responses in all the phenotypic domains.

In patients who do not achieve remission in all disease aspects on biologic monotherapy or combination of a biologic therapy with an oral synthetic agent; dual targeted anti-cytokines strategies or combined biologic with a targeted oral small molecule are a possible treatment option.

**Description/Method:**

A 40-year-old attended our rheumatology department in 2010 with peripheral synovitis, dactylitis and scalp psoriasis. A clinical diagnosis of PsA was made and she was commenced on combination methotrexate and sulphasalazine.

In 2011, therapy was escalated to include etanercept and methotrexate due to persistent active disease clinically and on nuclear medicine bone scan.

Between 2011 to 2015, sequential bDMARD switches including certolizumab, infliximab and adalimumab, failed to put the disease in remission.

Due to loss of effect, adalimumab was switched to secukinumab in early 2016. A few months later, while on secukinumab, she developed new onset Crohn’s disease and required treatment change to ustekinumab.

In August 2018 following a prolonged flare, ustekinumab was stopped and tofacitinib was started. The disease went in LDA until September 2020 when hospital admission for a severe PsA flare was required. She had dactylitis of 3 digits, a left temporomandibular joint effusion on ultrasound and elevated acute phase reactants. A treatment change to abatacept resulted in primary failure and abatacept was replaced with baricitinib.

In March 2021, she attended clinic with new nail changes, dactylitis of 3 digits and a large left knee effusion. She underwent a diagnostic arthroscopy of the left knee and wash-out in May 2021 which showed a macroscopic synovitis score and vascularity score of 100% respectively. Synovial histology showed evidence of chronic synovitis with abundant plasma cells.

Following the arthroscopy and careful discussion, it was decided to initiate combination therapy with adalimumab biosimilar and tofacitinib in June 2021. The patient was assessed at 2 months following starting combination therapy and a dramatic clinical and biochemical was noted. CRP had improved from 139.9mg/L to 6.3mg/L. She was mobilising without an aid for the first time in years. Both her psoriasis and Crohn’s disease were also well controlled.

**Discussion/Results:**

While the outcomes of patients with psoriasis have dramatically improved with monoclonal antibody therapies targeting IL-23 and IL-17A; achieving a measurable low disease activity state such as minimal disease activity (MDA) for musculoskeletal manifestations of psoriatic disease is infrequent. Due to the heterogeneity of disease in PsA, multiple cellular pathways are involved in the pathogenesis resulting in recalcitrant disease in a subgroup of patients. These patients, like our case, exhibit poor response to the current therapeutic paradigm in PsA.

In such patients, treating one disease domain, one type of cell or a single pathway at a time is unlikely to result in meaningful disease control and remission may be unachievable thus subjecting patients to sustained joint inflammation and damage seen in PsA.

Very little data exist regarding the safety of dual immunomodulator therapies for concomitant psoriasis and psoriatic arthritis. Safety concerns such as infectious risks are important considerations with such strategies; however, the targeted second-generation anti-cytokine biologics and targeted JAK-I have exhibited improved safety profiles with regards to infections.

In this case, following careful discussion with our patient, she felt that the prospect of possible reduction in her musculoskeletal symptoms outweighed the potential safety concerns.

Multiple cases are reported in the literature regarding the use combined biologic strategies in psoriatic disease; the most common combination reported being anti-TNF therapy and ustekinumab with varying degrees of response. Prospective, high-quality studies are needed to identify safe and efficacious dual strategies in PsA to pinpoint dosages, sequence and frequency of therapies.

**Key learning points/Conclusion:**

This case truly highlights the heterogeneity and complexity that accompanies psoriatic disease and as such, modulating a single pathway may not be sufficient to address all the phenotypic domains (skin, synovium, entheses, and axial).

While physicians are often, understandably, hesitant to prescribe combined immunomodulatory therapies due to concerns regarding potential complications, such strategies should be considered in debilitating cases of psoriatic disease who fail to respond to conventional treatment as they have the potential to foster deep and long-lasting remission.

Our case highlights that a patient-centred approach with patient involvement in clinical decisions regarding their disease is required. With such strategies, patients and clinicians, both, should balance the potential associated risks particularly infectious risk.

Further studies are required to better determine the efficacy and safety profile of combined immunomodulatory therapies in psoriatic disease.

